# Complement Inhibitors in Clinical Trials for Glomerular Diseases

**DOI:** 10.3389/fimmu.2019.02166

**Published:** 2019-09-27

**Authors:** Peter F. Zipfel, Thorsten Wiech, Ramona Rudnick, Sara Afonso, Fermin Person, Christine Skerka

**Affiliations:** ^1^Department of Infection Biology, Leibniz Institute for Natural Product Research and Infection Biology, Jena, Germany; ^2^Friedrich-Schiller-University, Jena, Germany; ^3^Institute of Pathology, University Hospital Hamburg-Eppendorf, Hamburg, Germany

**Keywords:** inhibitors, clinical trials, glomerular disease, C3 glomerulopathy, complement, ANCA, aHUS

## Abstract

Defective complement action is a cause of several human glomerular diseases including atypical hemolytic uremic syndrome (aHUS), anti-neutrophil cytoplasmic antibody mediated vasculitis (ANCA), C3 glomerulopathy, IgA nephropathy, immune complex membranoproliferative glomerulonephritis, ischemic reperfusion injury, lupus nephritis, membranous nephropathy, and chronic transplant mediated glomerulopathy. Here we summarize ongoing clinical trials of complement inhibitors in nine glomerular diseases and show which inhibitors are used in trials for these renal disorders (http://clinicaltrials.gov).

## Introduction

Defective complement action is a cause of several human glomerular diseases including atypical hemolytic uremic syndrome (aHUS), anti-neutrophil cytoplasmic antibody mediated vasculitis (ANCA), C3 glomerulopathy, IgA nephropathy, immune complex membranoproliferative glomerulonephritis, renal ischemic reperfusion injury, lupus nephritis, membranous nephropathy, and chronic transplant mediated glomerulopathy (1–3).

Pathology of these kidney disorders are caused or modified by (i) genetic alterations in complement genes that lead to impaired protein expression and/or function (4), (ii) by autoantibodies that target complement components or regulators, (iii) by autoantibodies which recognize specific surface structures, DNA and IgGs, and upon binding initiate complement or (iv) as recently shown by altered plasma levels of FHR-modulators (5). Understanding how the gene variants deregulate complement, or how disease relevant autoantibodies interfere in the cascade help to understand the underlying pathomechanisms of these renal disorders and allow to pinpoint for each disease which step and which level of the cascade is compromised or modified. This provides a rationale for treatment with complement inhibitors (5), and allows to address the questions, where and at which level the complement cascade should be targeted and for how long a complement inhibitor should be used.

The complement inhibitors Eculizumab (Soliris), Berinert, or Cinryze are currently approved by the Food and Drug administration (FDA) in the US and the European Medicines Agency (EMA). A new generation of complement inhibitors is currently evaluated in clinical trials and new inhibitors are being developed and tested in preclinical settings. Different types of inhibitors exist, including humanized monoclonal antibodies, small proteins binding to specific complement components, and furthermore recombinant proteins allowing substitution of defective or absent proteins, as well as a small interfering RNA.

Here we summarize the ongoing clinical trials of complement inhibitors in nine glomerular diseases and show which inhibitors are used in trials for these renal disorders (http://clinicaltrials.gov).

## Complement: Initiation, Major Enzymatic Check Points, and Multiple Effector Levels

**Figure 1:**
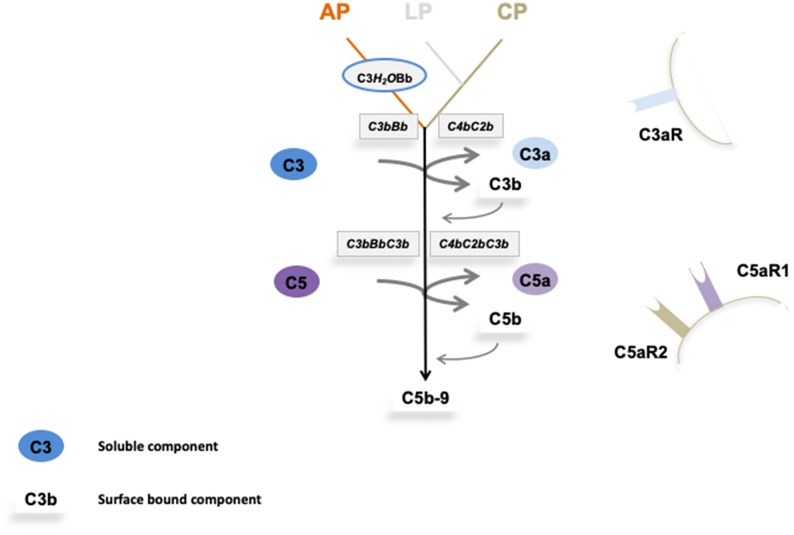
Overview on complement activation and cascade progression. Complement activation is mediated by the alternative pathway in the fluid phase and on surfaces, and by the lectin and the classical pathway on surfaces. Multiple regulators determine and adjust cascade progression and subsequence effector action. Thereby discriminating the action between intact self, altered self, and non-self. Three initial reactions activate the complement cascade and have different initial triggers. The alternative pathway (AP) which is initiated spontaneously and continuously in the fluid phase and in the absence of regulators is amplified on surfaces. Both the lectin (LP) and the classical pathway (CP) are initiated on surfaces. The type of surface influences activation and the regulator repertoire decides on cascade progression or inhibition. Different regulators control cascade progression in the fluid phase and on surfaces. The three pathways form surface bound convertases, the AP allows generation of the AP-C3 convertase and the LP/CP trigger CP convertase. The AP C3 convertase also triggers a potent amplification loop. The general role of both C3 convertase is to cleave the abundant plasma protein C3 (concentration 1,000–1,500 μg/ml) into the anaphylatoxin C3a and the opsonic C3b. The enzymatic response on the first enzymatic levels is frequently enhanced by the potent self-amplifying amplification loop. If activation C5 convertases are generated and again C5 convertases of the AP and of the LP/CP pathways do exist. The major role of the C5 convertase is to cleave C5 (plasma concentration 350 μg/ml) into the powerful anaphylatoxin C5a and to generate C5b. Surface bound C5b initiates terminal complement and formation of the terminal complex, C5b-9, also termed membrane attack complex which can form lytic pores. Thus, complement acts on two major enzymatic levels, each of which generates a unique set of effector components with rather diverse functions. The complexity of this cascade is mediated by regulators and inhibitors, which control activation in the fluid phase (AP) and which ensure that activation mainly occurs on non-self surfaces or modified surfaces. In the physiological setting this coordinated action allows to direct the toxic and clearance power to the foreign/modified particles. In case of any dysbalance this action can be targeted toward self-structures and this can cause pathology at specific sites.

To appreciate the many steps of complement activation and the fine balanced regulation an overview of this important homeostatic system is presented. The complement cascade has three activation routes, acts via two major enzymatic levels, which provide central checkpoints for the control of effector response and initiate the pore forming terminal complex (Figure 1). Overactive effector pathways drive and enhance specific responses that cause kidney pathology (6). The progression of each step and specific activation pathways are regulated by selectively acting endogenous inhibitors and this control provides the rational to where and at which level activation and pathway progression can be influenced by therapeutic agents.

Initial spontaneous activation of the complement cascade by the alternative pathway (AP) occurs in the fluid phase, activation proceeds and propels on surfaces. The lectin (LP) and the classical pathway (CP) are initiated on target surfaces by different recognition and initiator proteins (7, 8). Upon activation two central enzymatic levels are formed, both of which cleave a soluble complement compounds C3, C4, and C5, and generate small, soluble and cell attracting inflammatory mediators, i.e., C3a, C4a, and C5a and also form the surface acting effector compounds, C3b, C4b, and C5b. The first enzymatic level is mediated by two newly formed C3 convertases with overlapping activity (9). Both the AP and the LP/CP convertase (C3bBb and C4bC2b, respectively) cleave the central complement protein C3 and generate the soluble inflammatory mediator C3a and surface acting C3b which opsonizes target surfaces (10). In addition this level can generate a potent, self-amplifying loop which enhances and propels C3 conversion, generates more effector products and thereby increases the density of deposited C3b can subsequently be processed by specific proteases which are assisted by cofactors and regulators. The processed variants, iC3b, C3dg, or C3d are recognized by different receptors and mediate important effector functions (6).

Subsequently when activation proceeds a second enzymatic level is formed. Two C5 convertases, i.e., the AP pathway (C3bBbC3b) and the LP/CP pathway (C4bC2bC3b) convertases, which use C5 as substrate are generated. C5 cleavage produces the potent soluble anaphylatoxin C5a and C5b is deposited on the target surface (10). Surface deposited C5b initiates the third major effector part and forms the pore forming terminal complex C5ab-9, also termed TCC (terminal complement complex), which inserts in the target membrane, forms a pore and causes cell lysis (Figure 1).

Thus, complement when activated generates several effectors. The major and primary soluble effectors are the anaphylatoxins C3a, C5a, and C4a that by recruiting and activating immune cells, induce complement inflammation. Recently PAR1 (protease-activated receptor) and PAR4 were identified as non G-coupled receptors for C4a and a FHR1 receptor in form of EMR1 was reported (11, 12). The major surface acting effectors are C3b, the C3- and the C5 convertases and C5b-9. C3b deposition results in opsonophagocytosis and C5b-9 deposition induces the pore forming, membrane damaging C5b-9 complex, and soluble C5b-9 likely has pro-inflammatory activity (1, 2).

Complement action can initiate in the fluid phase and on surfaces and a large panel of regulators controls cascade progression at many sites and in specific steps. Regulators which are distributed in the fluid phase or are expressed on cellular surfaces control initiation of each activation pathway, formation and action of the C3 convertases, density and type of deposited C3b, the fate of C3b, formation and action of the C5 convertase, fate and half-life of the anaphylatoxins and formation of the terminal complement complex.

An important challenge is to understand complement regulation, define how the activators and the many regulators cooperate with each other, understand which inhibitor acts at which site and furthermore how and when the absence of one single regulator, or a single defective regulator affects the concerted action and disturbs the cascade. Understanding of the homeostatic role of this evolutionary old system, and characterizing the precise action of each single regulator and their interplay is highly relevant to elucidate the pathological principles of complement associated disorders. This ultimately allows to design precisely acting complement targeting therapeutic agents.

## Diseases

### Hemolytic Uremic Syndrome (HUS)

*HUS*, hemolytic syndrome (HUS) is characterized by an over activated complement system. This disease which has different triggers is defined by hemolytic anemia, thrombocytopenia and acute renal damage resulting in thrombotic microangiopathy (TMA). The majority of HUS cases are caused by infections, with enterohemorrhagic, shiga toxin producing *Escherichia coli* (EHEC*)*, inducing Shigatoxin HUS. Similarly, infections with *Streptococcus pneumoniae* can cause pneumococcal HUS. HUS induced by infections is more frequent in children (13, 14).

Genetic alterations and autoantibodies cause very similar or even the same clinical symptoms. Genetic-atypical HUS, accounts for about 15% of total HUS cases and is more frequent in adults. About 60–70% of these patients have genetic defects in one or several genes, which include nine complement genes (15–17) and a gene coding for a cytoplasmic signaling protein (18). The affected genes encode proteins, that (i) form the alternative pathway C3 convertase (C3, Factor B), (ii) regulate the activity of this central complement enzyme (Factor H, MCP/CD46, Factor I), (iii) act as complement modulators (FHR1, FHR3, FHR4, thrombomodulin) (18), or (iv) the cytoplasmic signaling protein (DAGKε) (19). This puts local AP C3-convertase induced complement and C3 regulation in the focus of genetic aHUS mediated pathology and shows the important role of the lytic branch of complement.

An autoimmune form of HUS is observed in about 15% of HUS patients (20, 21). Most autoantibodies have the same binding profile; they bind to the C-terminal recognition region of Factor H (SCRs19-20) and block Factor H surface binding. Most HUS patients with autoantibodies are of young age and have a homozygous deletion of a ca 24 kb chromosomal segment which encompasses the *CFHR3-CFHR1* genes. Therefore, this form is also termed DEAP-HUS patients (**DE**ficient for FHR1-FHR3 and **A**utoantibody to Factor H **P**ositive).

**Figure 2:**
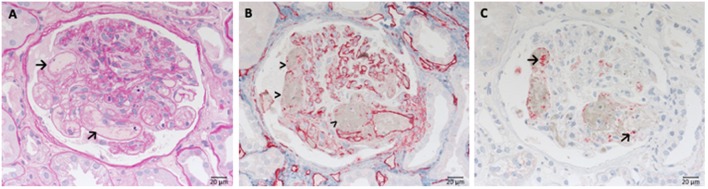
Morphological changes in atypical uremic hemolytic syndrome (aHUS) resulting in thrombotic microangiopathy (TMA). **(A)** Periodic acid Schiff (PAS) reaction: focal loss of endothelial cells and fibrin precipitates in dilated glomerular capillaries (→). **(B)** Loss of CD34-positive endothelial cells stained red (>). **(C)** Accumulation of platelets stained red in CD61 immunohistochemistry (→).

Due to the over activation of the complement system the endothelial cells of glomerular capillaries are damaged or destroyed (Figures 2A,B) resulting in the formation of the typical microscopic thrombi of acute TMA cases (Figures 2A,C). As a result of multiple thrombus formation in TMA glomerular sclerosis might arise. After a possible resolution of the initial TMA episode, the glomerular capillaries can be remodeled. These activated endothelial cells might synthesize a new glomerular basement membrane (GBM) resulting in a GBM duplication or multilayering, typically found in chronic or relapsing TMA. An overview of typical histological features of genetic aHUS induced acute TMA is displayed in Figure 2.

Genetic aHUS is a prime example of a complement mediated disease and the terminal complement inhibitors Soliris/Eculizimab is already approved and on the market for therapy. This monoclonal antibody binds to C5, blocks C5 activation and formation of the terminal complement complex, i.e., C5b-9. Eculizumab was approved for treatment of genetic aHUS by the Food and Drug Administration (FDA) and the European Medicines Agency (EMA) in 2011. Currently a new version of this C5 binding drug is in clinical trials for genetic aHUS. Ravulizumab (or ALXN 1210), is a pH dependent, C5 binding antibody which releases C5 at low pH. This drug is already approved for PNH (2018) (22–25) (Table 1). For genetic aHUS additional C5 targeting complement inhibitors are evaluated in clinical studies. These include the lectin pathway inhibitor OMS721, which binds and blocks MASP2 action, is evaluated in phase III, coversin, a tick (*Ornithodoros moubata)* derived C5 inhibitor and CCX168 the C5aR1 antagonist in phase II studies (Table 1). Studies with ALN-CC5 (Cemdisiran), a siRNA which blocks hepatic C5 production were performed, but apparently this trial was withdrawn.

**Table 1:** Complement inhibitors being evaluated in clinical trials of glomerular diseases.

**Disease**	**Inhibitor name**	**Alternative name**	**Inhibitor type**	**Inhibitor target**	**Company**	**Phase**	**Clinical trial code**	**Comments**
aHUS	OMS721	OMS 00620646	Antibody	MASP2	Omeros	III	NCT03205995	
	Eculizumab	Soliris	Antibody	C5	Alexion	**Market**		NCT02574403; Phase 4, duration of Eculizumab treatment
	Ravulizumab	ALXN1210	pH-dependent Antibody		Alexion	III	NCT03131219 NCT02949128	Ravulizumab is already approved for paroxysmal nocturnal haemoglobinuria
	Coversin (rVA576)	Nomacopan	Peptide		Akari Therapeutics	III	NCT03829449	
	CCX168	Avacopan	Small molecule	C5aR1	ChemoCentryx	II	NCT02464891	
ANCA-associated vasculitis	IFX 1	CaCP 29	Antibody	C5a	InflaRx	II	NCT03712345	Granulomatosis with Polyangiitis; Microscopic Polyangiitis
	CCX168	Avacopan	Small molecule	C5aR1	ChemoCentryx	III	NCT02994927	
C3 Glomerulopathy	OMS721	OMS 00620646	Antibody	MASP2	Omeros	II	NCT02682407	DDD
	AMY-101		Antibody	C3	Amyndas	I	NCT03316521	
	APL-2		Peptide		Apellis	II	NCT03453619	DDD and C3 glomerulonephritis
	ACH 4471	ACH-0144471	Small molecule	FD	Achillion	II	NCT03459443	DDD and C3 glomerulonephritis
							NCT03369236	DDD and C3 glomerulonephritis
							NCT03124368	DDD and C3 glomerulonephritis
	LNP023		Small molecule	FB	Novartis	II	NCT03832114	C3 Glomerulonephritis
	Eculizumab	Soliris	Antibody	C5	Alexion	I	NCT01221181	DDD and C3 glomerulonephritis
						II	NCT02093533	C3 glomerulonephritis
	CCX168	Avacopan	Small molecule	C5aR1	ChemoCentryx	II	NCT03301467	DDD and C3 glomerulonephritis
IgA nephropathy	OMS721	OMS 00620646	Antibody	MASP2	Omeros	III	NCT03608033	
	APL-2		Peptide	C3	Apellis	II	NCT03453619	
	LPN023		Small molecule	Factor B	Novartis	II	NCT03373461	
	Cemdisiran	ALN-CC5	RNAi	C5	Alnylam	II	NCT03841448	
	CCX168	Avacopan	Small molecule	C5aR1	ChemoCentryx	II	NCT02384317	
Immune complex membranoproliferative glomerulonephritis	ACH 4471	ACH-0144471	Small molecule	Factor D	Achillion	II	NCT03459443 NCT03124368	
Ischemic reperfusion injury	C1INH	Berinert	Protein	C1r and C1s	CSL Behring	I	NCT02134314	C1INH is already approved and on the market for hereditary angioedema
Lupus nephritis	OMS721	OMS 00620646	Antibody	MASP2	Omeros	II	NCT02682407	
	APL-2		Peptide	C3	Apellis	II	NCT03453619	
Membranous nephropathy	OMS721	OMS 00620646	Antibody	MASP2	Omeros	II	NCT02682407	
	APL-2		Peptide	C3	Apellis	II	NCT03453619	
Transplant	C1INH	Cinryze	Protein	C1r and C1s	Shire	III	NCT02547220	Acute Antibody-Mediated Rejection(for patients with kidney transplant)
		Berinert			CSL Behring	I	NCT02134314	ESRD
							NCT01134510	Kidney transplant - therapy to prevent organ rejection
	AMY-101		Antibody	C3	Amyndas	I	NCT03316521	
	Eculizumab	Soliris	Antibody	C5	Alexion	II	NCT02145182	Prevention of delayed graft function
	LFG-316	Tesidolumab	Antibody		Novartis	I	NCT02878616	ESRD

The overall experience with Eculizumab is very good and positive results have been reported for long-term therapy. However, also incomplete inhibitory activities are reported e.g., for patients with C5 mutations (26). It will be of interest to compare how different targeting strategies, e.g., directly targeting C5, the lectin pathway, or the C5a receptor effector pathway influence the outcome and if genetic aHUS patients with different gene mutations or autoantibodies respond differently to the various inhibitors.

### ANCA Associated Glomerulonephritis

ANCA (anti-neutrophil cytoplasmic antibody-) associated vasculitis (AAV) describes a collection of related disorders, which include granulomatosis with polyangiitis (GPA), microscopic polyangiitis (MPA), and eosinophilic granulomatosis with polyangiitis (EGPA), also called Churg-Strauss syndrome (27). A characteristic feature of these diseases are autoantibodies which cause complement inflammation and cell infiltration in blood vessels and which result in necrosis. The pathological principle of the autoantibodies is targeting neutrophil proteins myeloperoxidase and proteinase 3. AAV affects approximately 40,000 people in the US and approximately 4,000 new cases are identified each year. In Europe more than 75,000 people are affected and at least 7,500 new cases emerge each year (28).

Complement inflammation triggered by the alternative pathway is associated with ANCA. Autoantibodies induced C5a generation leading to neutrophil infiltration and activation, which initiates a vicious cycle: more neutrophils are attracted to local sites and the activated cells release their granule contents. The damage occurs in small blood vessels, mostly in the kidney but also other organs like lungs, nerves and sinuses can be involved (24).

**Figure 3:**
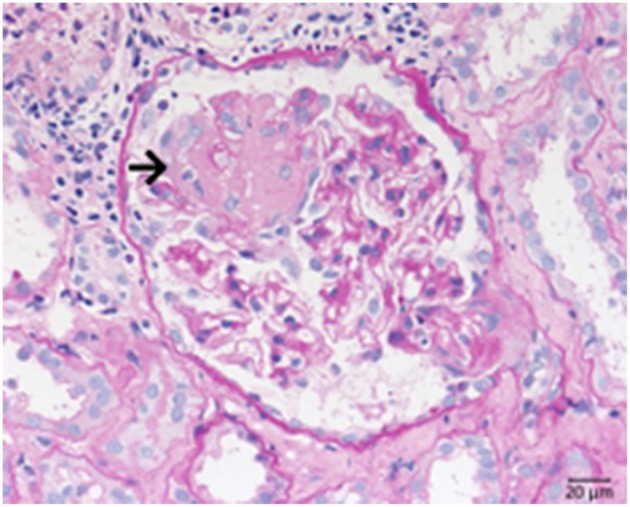
Morphological changes in ANCA associated pauci-immune necrotizing glomerulonephritis. PAS-reaction of a case of ANCA-associated Glomerulonephritis, displaying a rupture of the glomerular basement membrane. See fresh fibrin precipitates inside the bowman space at the side of the necrosis (→).

Because of the damage caused by the degranulation of granulocytes necrosis and subsequent rupture of small vessels arise (Figure 3), particularly inside glomerular tufts. This is followed by proliferation of parietal cells and influx of macrophages, leading to crescent formation, and later segmental (partial) or complete scarring of the affected glomeruli occurs. Due to the almost complete lack of immune deposits inside of the glomeruli the renal manifestation of ANCA associated diseases is frequently called pauci-immune necrotizing glomerulonephritis. However, contrary to the assigned name complement molecules have been detected inside glomeruli affected by AAV in immunohistochemical and mass spectrometry-based studies. The typical picture of a fresh necrosis of glomerular capillaries in AAV is shown in Figure 3.

These pathological aspects make the C5a–C5aR1 axis of particular interest for therapeutic intervention in order to block attraction of neutrophils to local sites, inhibit cell activation and vascular destruction. Ongoing clinical trials approach the inflammatory complement C5a–C5a receptor 1 (C5aR1) axis with the soluble C5a peptide binding mAb (IFX-1; InflaRx) in phase II and the C5a receptor antagonist (Avacopan, Chemocentryx) in phase III trials. A clinical trial with Eculizumab was initiated and but enrollment of patients was terminated (Table 1).

### C3 Glomerulopathy With Membranous Nephropathy and Dense Deposits Disease

**Figure 4:**
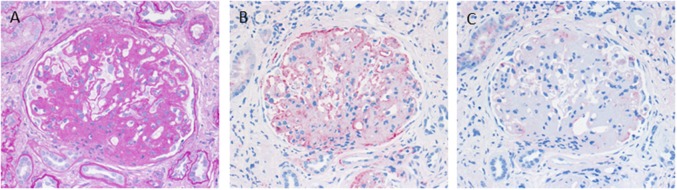
Morphological appearance of C3 glomerulopathy. **(A)** PAS reaction reveals a membranoproliferative pattern with double contours of the GBM. **(B)** Strong immunohistochemical positivity for C3 at the GBM and in the mesangium. **(C)** Only very scant and segmental positivity for IgG.

**Figure 5:**
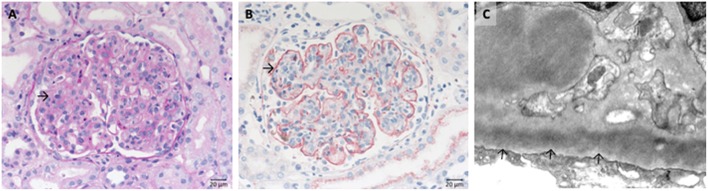
Morphological changes in dense deposit disease (DDD). **(A)** PAS–reaction of a dense deposit disease case. Note the mesangial and endocapillary hypercellularity without prominent double contours of the glomerular basement membrane (→). **(B)** Note a strong red positivity for C3 inside the glomerular basement membrane (→). **(C)** The name giving electron dense deposits within the thickened glomerular basement membrane (GBM,→).

C3 glomerulopathy is an umbrella term for a spectrum of related diseases. The diagnosis is primarily based on evaluation of renal biopsies showing prominent immunofluorescent or immunohistochemical staining for C3, which should be two orders of magnitude more intense than staining for immunoglobulins like IgA, IgG, or IgM (29, 30). The major subtypes of C3 glomerulopathy are C3 dominant membranoproliferative glomerulonephritis (C3 MPGN) (Figure 4) and dense deposit disease (DDD) (Figure 5). DDD is identified by electron microscopy showing thickened glomerular basement membranes with very electron dense material within the membrane and in the mesangium accompanied by mesangial proliferation (31) (Figure 5). Due to historic reasons, DDD is sometimes still referred to as MPGN II. In C3 glomerulopathy cases displaying MPGN patterns, double contours of the GBM as well as endocapillary and mesangial hypercellularity arises (32) (Figure 5).

C3 glomerulopathy is caused by defective complement regulation with genetic, as well as autoimmune causes. Often defective complement regulation occurs in plasma in the fluid phase and correlates with C3 consumption and low C3 plasma levels. Other forms of this disease develop on basis of normal plasma C3 levels. Genetic causes of C3 glomerulopathy include mutations in the genes coding for *Factor H, C3, CFHR1, CFHR2, CFHR3, CFHR5*, and Factor B. *Factor H* gene mutations are mostly homozygous or compound heterozygous. In C3 glomerulopathy multiple complex patterns of *CFHR* gene variations are reported. Alterations include single nucleotide variations in one of the five *CFHR-*gene, and structural variations in the *CFHR* gene cluster with duplications and deletions of gene elements or of larger chromosomal segments. These genetic alterations generate FHR variant proteins, FHR hybrid proteins and furthermore alter the FHR plasma levels.

**Autoimmune factors in C3 glomerulopathy** include autoantibodies in form of C3-Nephritic Factor, C4-Nephritic Factor or C5-Nephritic Factor. Most autoantibodies bind to neoepitopes exposed in these central complement enzymes, however some autoantibodies also bind to the single components, i.e., C3, C3b, Factor H or Factor B. These different autoantibodies show that complement action on the level of the C3 convertase, as well as C5 convertase are relevant for this disease spectrum (33–35).

Investigator initiated trials with C5 targeting by Eculizumab were reported for C3 glomerulopathy. First treatments showed favorable outcome, however studies with large patients cohorts revealed positive effects of Eculizumab in a fraction, about 40% of the patients, but not in all patients (36–38).

Currently seven complement inhibitors are evaluated in clinical phase I/II trials: OMS721, the MASP2 inhibitor (Omeros), Amy 101 (Amyndas), APL2 a C3 targeting peptide (Apellis), ACH-4471, a Factor D binding antagonist (Achillion), LNP023 a Factor B blocking compound (Novartis), Eculizumab (Alexion), and the C5a receptor 1 targeting Avacopan (Chemocentryx) (Table 1).

ACH-4771 is a small Factor D inhibitor that is applied orally and that blocks the catalytic side of Factor D. In presence of inactive Factor D the alternative pathway convertase C3bBb, is not formed and complement activation does not proceed. The other orally administered inhibitor LPN023, binds to the active site of Factor B, inhibits the alternative pathway C3 convertase and blocks C3 cleavage. Thus, different inhibitors are currently evaluated which target different levels of the complement cascade, the activation level, the lectin pathway, the C3 convertase of the AP, C3- and C5 cleavage. In this regard, C3 glomerulopathy has the potential to develop to an example for a disease where complement therapy will be approached based on personalized gene or autoimmune profile. Based on the different action sites of the inhibitor, it will be of interest to see which compound or which targeted pathway is most effective and which subform responds or benefits from which inhibitor.

### IgA Nephropathy

IgA nephropathy (IgAN) is a leading cause of chronic kidney diseases with a complex disease pathology and with several factors involved (39). Genome–wide association studies identified the *CFHR*-gene cluster as a susceptibility locus and opposing effects were reported for individual *CFHR* genes (40). Homozygous *CFHR1/CFHR3*-deficiency resulting in the absence of FHR1 and FHR3 in plasma is protective and *CFHR5* is an IgAN susceptibility gene (41–44). Rare FHR5 protein variants with altered C3b binding represent risk factors (44). Current work focuses on FHR1- and FHR5-plasma levels (45, 46). Elevated FHR1- plasma levels and higher FHR1::Factor H-ratios influence alternative pathway regulation and correlate with disease severity (45–47). In addition also variations of FHR-plasma levels are of pathological relevance (48–50) and enhanced FHR5 plasma levels is an independent risk factor (46). Altered FHR1 and FHR5 plasma levels, or FHR1/Factor H ratios are disease relevant are related to diseases severity and correlate with alterations in glomerular filtration rates (49–51).

Autoimmune factors include galactose deficient IgA immune complexes or autoantibodies against O-glycans and C3. Genetic and clinic studies link complement and the alternative pathway to immune pathogenesis of IgA nephropathy. But also environmental factors play a role for diseases development. In addition properdin, a complement activator and complement Factor H are identified in the immune deposits.

**Figure 6:**
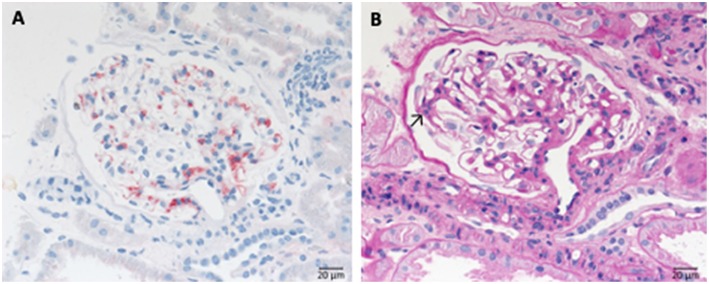
Morphological changes in IgA nephropathy: **(A)** IgA immunohistochemistry of a case of IgA nephropathy, displaying a noticeable mesangial positivity. **(B)** PAS reaction of the same glomerulus: note the mesangial matrix increase and the focal mesangial hypercellularity (arrow).

IgA nephropathy is marked by the deposition of galactose-deficient IgA1 in the glomerular mesangium (Figure 6A). As a result, mesangial matrix increase and some proliferation of mesangial cells is often seen (Figure 6B), moreover, endocapillary hypercellularity, and eventually rupture of glomerular capillaries with subsequent formation of crescents might occur (extracapillary component). Depending of the severity of the disease glomerular and interstitial injury arises. However, clinical course of IgA nephropathy varies from almost absent clinical symptoms in many cases to fulminant renal failure resulting in dialysis dependent loss of renal function within weeks. Numerous studies suggest that the histological pattern and the deposition of complement components such as C1q, C3c, and lectin are predictors of worse outcome. The classical appearance of mesangioproliferative IgA nephropathy is depicted in Figure 6.

Clinical studies addressing the complement system in IgA Nephropathy include targeting the MASP-2 by OMS721 (Omeros) (phase III), C3 by APL-2 (Apellis), Factor B by LPN023 (Novartis), C5 by Cemdisiran, and C5aR1 by CCX168 (Chemocentryx) (Table 1).

### Immune Complex Membranoproliferative Glomerulonephritis

**Figure 7:**
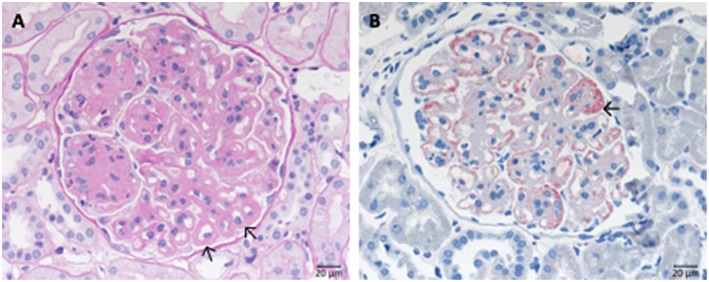
Morphological changes in immune complex membranoproliferative glomerulonephritis (MPGN). **(A)** PAS-reaction of a case of membranoproliferative glomerulonephritis due to a chronic hepatitis C infection. Note the lobular appearance and double contours of the GBM (→). **(B)** Note positivity for IgG at the side of double contours of the GBM (→).

Immune complex membranoproliferative glomerulonephritis (MPGN) is marked by dominant deposition of immunoglobulins (Figure 7) and to a lesser extent of complement components inside the mesangium and along the inner side of the GBM. As a result, mesangial, and endocapillary hypercellularity as well as GBM duplication arise (Figure 7B). Additionally, necrosis and crescent formation might occur. Furthermore, in cryoglobulin associated cases hyaline pseudothrombi can be observed inside glomerular capillaries. Several putative causes for the development of immune complex MPGN are known, such as chronic infections, like endocarditis, monoclonal gammopathy, hepatitis C and the formation of cryoglobulins. An example of the typical histological pattern of immune complex MPGN is displayed in Figure 7.

Clinical trials in Immune complex membranoproliferative Glomerulonephritis are with ACH 4471 (Achillion) in phase II (Table 1).

### Lupus Nephritis

Lupus nephritis is an inflammatory kidney disease occurring in patients suffering from systemic lupus erythematosus (SLE) and active complement is important for the pathogenesis. The classical complement pathway, in particular the components C1, C4, and C2 were for a long time associated with this common form of kidney damage, in particular by defective removal or clearance of damaged self-cells, debris material, and immune complexes. Accumulated self-material initiate complement. In addition a role of the lectin and alternative complement pathway are shown in lupus nephritis (52, 53). Inflammatory complement initiated via C5a results in neutrophil infiltration, and autoantibodies to intracellular proteins induce the cascade further.

**Figure 8:**
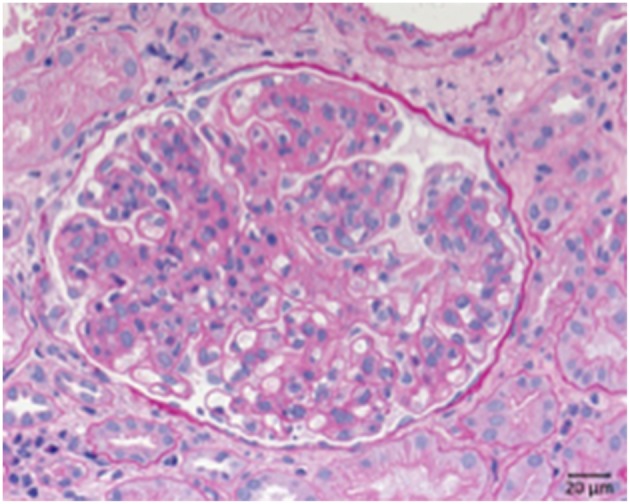
Morphological changes in lupus nephritis, class IV. PAS reaction of a lupus nephritis class IV. Note lobular pattern with pronounced endocapillary and mesangial hypercellularity as well as thickened GBM.

Systemic lupus erythematosus is an autoimmune disease usually involving multiple organs and as a possible complication the kidneys can be involved. The most important renal consequence of SLE is the development of immune complex glomerulonephritis. However, other possible damage patterns are TMA, interstitial nephritis, podocytopathy, and amyloidosis (Figure 8). SLE associated glomerulonephritis is separated according to the histologic appearance into six different classes (RPS/ISN). Class I lupus nephritis resembles glomerular IgG deposition without further morphological findings at the light microscopic level. Class II shows deposition of immune components, particularly IgG inside the mesangium. A mesangial proliferation is visible by light microscopy. Class III lupus nephritis shows additionally duplication of the GBM and/or endocapillary hypercellularity and/or crescent formation in a minority of the affected glomeruli. Class IV (Figure 8) is characterized by an MPGN like pattern with duplication of the GBM and/or endocapillary hypercellularity and/or crescent formation in the majority of glomeruli. Class V lupus nephritis resembles membranous glomerulonephritis and can occur in combination with any of the other mentioned classes or alone. As an end stage of lupus nephritis class VI is defined as scarring of over 90% of the sampled glomeruli inside a renal biopsy.

Genetic studies showed that the *CFHR1-CFHR3* deletion presents with increased risk for SLE (54). Homozygous deletion of this 24 kbp chromosomal *CFHR1-CFHR3* containing fragment has kaleidoscope features. First the deletion is common in the healthy population and shows different frequencies in ethnic groups, i.e., being present in ca 30% of the healthy African, 18% of the healthy Asian and 5–6% of the healthy European population (55). Second this homozygous setting confers risk for two renal diseases, SLE and DEAP-HUS and for infections with the pathogenic bacterium Neisseria meningitides. Third the same deletion has protective roles in IgAN, and in the retinal disease Age related macular degeneration (AMD).

In lupus nephritis two phase II clinical trials are ongoing with the complement inhibitors OMS 721 (Omeros) which targets the lectin pathway via MASP2 protein and APL2 (Apellis) which interferes with complement activation at the level of the central complement component C3 (Table 1).

### Membranous Nephropathy

Membranous nephropathy results from binding of IgGs to antigens expressed at the surface of podocytes. Membranous nephropathy is grouped into primary cases (80% of cases) without causative autoimmune disease (56, 57). In primary membranous nephropathy cases about 70% show autoantibodies directed against phospholipase A_2_ receptor 1 (PLA_2_R1), display antibodies directed against thrombospondin type 1 containing 7A (THSD7A) in serum (1–2%) and to complement Factor H (3%) (57–63). PLA_2_R and THSDA7 are podocyte antigens. The autoantibodies are directed to the receptors exposed on the surface of podocytes. Animal studies show that proteinuria is caused by the terminal complement complex. Also other mechanisms have been proposed.

**Figure 9:**
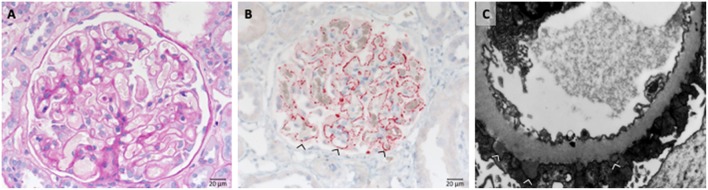
Morphological changes in membranous glomeruloneprhits nephropathy. **(A)** PAS-reaction with slightly thickened GBM. **(B)** PLA_2_R1 immunohistochemistry of a PLA_2_R1-antibody associated case of membranous glomerulopathy. Note strong granular positivity for PLA_2_R1 at the GBM (>). **(C)** Electron microscopic depiction of subepithelial electron dense deposits (>).

Also secondary cases (20% of cases) are known with causative diseases like SLE (Lupus nephritis class V). Complexes formed by the autoimmune IgG and the proper antigen are deposited along the outside of the GBM at the anchoring side of the foot processes of the podocytes (Figure 9).

Most scenarios of primary membranous nephropathy are mediated by autoantibodies to M type Phosphoplipase A_2_ receptor (PLA_2_R) (95%) and Thrombospondin type 1 domain containing 7a (THSD7a) receptor, a podocyte antigen (3–5%). Recently an additional third autoimmune form was described, where autoantibodies developed which target complement Factor H (3%) (63).

Most PLA_2_R and THSD7A autoantibodies are of the IgG4 subtype, a subtype which does not activate complement, however additional autoantibody forms are identified (56). Components of the classical and the alternative pathways are prominently localized at the site of the IgG-antigen deposits. The IgG-antigen complexes can be found along the outside of the GBM at the anchoring side of the podocyte foot processes (Figure 9B). The GBM expands and overgrows during disease development (Figure 8). Also older deposits inside the GBM are resorbed over longer time periods. As a result foot process retraction arises and nephrotic range proteinuria develops. The typical morphological changes in membranous nephropathy are shown in Figure 9C.

About 3% of MN patients have Factor H binding antibodies which target the C terminal recognition region of the human regulator and block surface binding (63). In the autoimmune form DEAP-HUS a related pathologic principle, with autoantibodies targeting the C -terminal binding region Factor H block Factor H surface action (64, 65), These features indicate that complement regulation and furthermore cell damage mediated by the terminal complement pathway is a disease relevant mechanisms at the podocyte surface.

Two complement inhibitors OMS 721 (Omeros), which targets the lectin pathway protease MASP2 and APL2 (Apellis), which binds to C3 component, respectively are currently evaluated in clinical trials of membranous nephropathy. Both are in phase II (Table 1).

### Renal Transplant

Complement is activated in transplanted organs and in particular a transplanted kidney can be challenged by activated complement. Numerous approaches are being used to limit complement activation in the transplant in order to block complement inflammation and complement terminal pathway action (66). Involved in renal transplant as the new, foreign surface and under resourced tissue provide a platform for complement activation (67).

**Figure 10:**
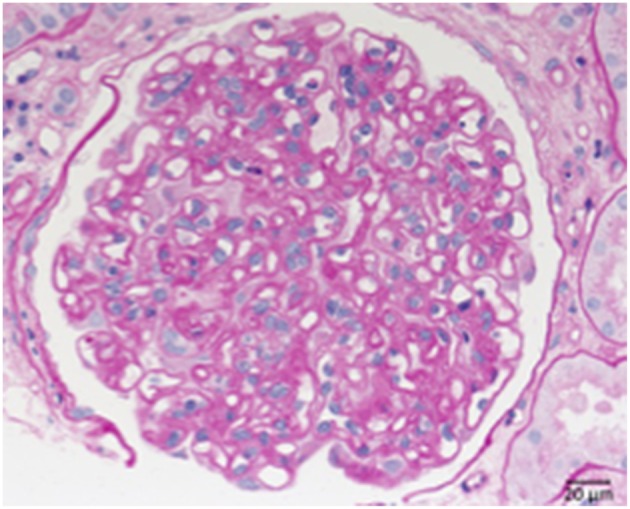
Chronic transplant glomerulopathy. PAS: Chronic transplant glomerulopathy displaying pronounced double contours of the GBM and slight endocapillary hypercellularity.

Two primary immunological damage mechanisms occurring in transplant kidneys are humoral rejection, with the formation of antibodies against structures of the transplanted organ and cellular rejection with the sensitization of T-cells against donor kidney antigens. The role of the complement system is particularly prominent in cases of humoral rejection. In these cases C4d, an indicator of acute humoral rejection can be deposited alongside peritubular capillary walls. In case of chronic humoral rejection the steady binding of antibodies leads to the activation or loss of endothelial cells particularly in glomerular and peritubular capillaries. This is followed by the formation of new GBM material (Figure 10). Therefore, a membrane duplication or multilayering of the basement membranes arise in glomerular and peritubular capillaries. The typical morphology of chronic transplant glomerulopathy is displayed in Figure 9.

Different clinical conditions are evaluated in form of acute antibody mediated rejection and also in end stage renal diseases and kidney transplantation to prevent organ rejection. Currently one phase III and two phase I studies are ongoing which evaluate efficacy of the C1 Inhibitors (Cinryze and Berinert). In addition the C5 inhibitor Eculizumab (Alexion) is tested in phase II, and the C5 monoclonal antibody LFG-316 developed by Novartis is tested in a phase I studies (Table 1).

#### Renal Ischemic Reperfusion Injury

Ischemic reperfusion injury is a common cause for acute kidney damage which can follow transplantation and which can damage the transplanted organ (68). Acute tubular damage is a common cause for kidney failure and the complement system which is activated on damaged self-cells can propel and increase such local damage (69). Ischemic reperfusion injury is marked by damage to the tubular epithelium. The tubular epithelial cells appear flattened or swollen and the cells suffer a total or partial loss of the brush border.

Complement inhibition in this form of kidney damage is being pursued with the C1 Inhibitor (Table 1).

## Summary Outlook

As outlined here, the current development shows that complement inhibition in renal disease is actively pursued in several clinical studies. Initial proof of concept comes from the inhibitor Soliris/eculizumab which is approved for treatment of genetic aHUS, as well as for two other complement disorders paroxysmal nocturnal hemoglobinuria (PNH) and myasthenia gravis (MG). The expanding list of trials and the increasing number of complement inhibitors, which are being developed and are tested in preclinical studies demonstrate that complement inhibition is an option for therapy of glomerular disorders.

**Figure 11:**
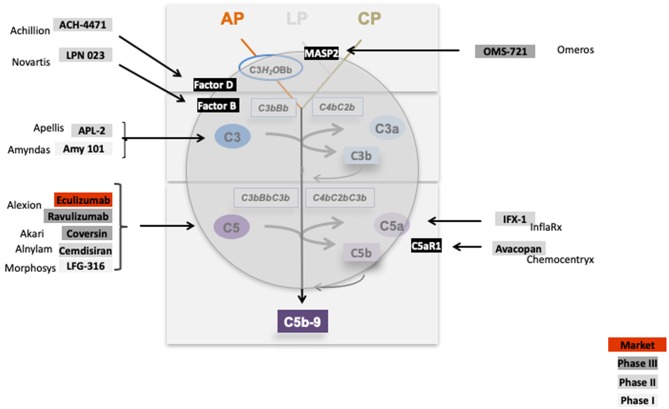
Complement inhibitors target different levels and steps of the complement cascade. Complement inhibitors which are evaluated in clinical trials for various kidney diseases bind to different complement proteins and inhibit the cascade at different levels. C1 inhibitor binds to C1 and blocks C1 activation. OMS 721 binds to the lectin pathway protease MASP2. C3 targeting proteins include APL2 (Apellis) and AMY 101 (Amyndas). The Factor D inhibitor ACH-4471 (Achillion) binds to Factor D, a protease which cleaves in its active state Factor B. LPN023 (Novartis) a small Factor B binding protein blocks formation of the enzymatically active AP C3 Convertase. Several compounds target complement at the level of C5. Eculizumab, and the new version Ravulizumab (both Alexion) bind to C5 and block activation of the protein. Coversin is a tick derived C5 binding protein (Akari) and C5 inhibitor. C5 synthesis is blocked by Cemdisiran as an RNAi targeted strategy (Alnylam), and by LFG-316 (Novartis). The complement inflammatory C5a—C5aR1 axis is inhibited by IFX-1 (InflaRx) and Avacopan (Chemocentryx).

The inhibitors which are tested in clinical trials for glomerular diseases target the activation pathways i.e., the lectin pathway via MASP-2, the central component C3 (APL1 and Amy101), the alternative pathway convertase via Factor D (ACH4471), Factor B (LPN 023), target the terminal pathway via C5 (Eculizumab, LFG-316), by blocking C5 synthesis in the liver via C5-siRNA (Alnylam), or directly interfering with the inflammatory C5a—C5aR1 axis (IFX1; InflaRX, and Avacopan; Chemocentryx). Thus, these complement inhibitors target different proteins in different activation pathways or effector levels of complement (Figure 11). This provides the option to block complement at different levels and to interfere with different effector pathways. Given that complement is involved in many renal (and also other) diseases the existing inhibitors allow interfering with complement at different stages.

These inhibitors target complement at different levels and address the various effector pathways. A detailed understanding of the pathological mechanism for each single disease and also of the subforms, combined with an understanding of the mode of action of each inhibitor and a better understanding of the regulatory loops, regulatory networks and feedback pathways of the complement cascade, as well as the crosstalk with other immune systems like the coagulation cascade will allow to use the inhibitors for the clear benefit of the patient. It will be of interest to see which of the various inhibitors is most effective for the outlined renal diseases and given the heterogeneity of the diseases and their existing subforms it will be of interest to evaluate the different causes and responses of the inhibitors. In total, many clinical trials and the emerging list of additional new inhibitors show that complement inhibition in glomerular diseases has a very promising future.

## Author Contributions

PZ, TW, and CS designed the concept and planned the work. RR, SA, and FP performed the work. All authors wrote the manuscript.

### Conflict of Interest Statement

The authors declare that the research was conducted in the absence of any commercial or financial relationships that could be construed as a potential conflict of interest.
